# Intra-household evaluations of alcohol abuse in men with depression and suicide in women: A cross-sectional community-based study in Chennai, India

**DOI:** 10.1186/s12889-015-1864-5

**Published:** 2015-07-11

**Authors:** Adyya Gupta, Bavani Priya, Joseph Williams, Mona Sharma, Ruby Gupta, Dilip Kumar Jha, Shah Ebrahim, Preet K. Dhillon

**Affiliations:** South Asia Network for Chronic Disease (SANCD), Public Health Foundation of India, National Capital Region, India; Voluntary Health Services, Chennai, India; Centre for Mental Health, Public Health Foundation of India, National Capital Region, India; Department of Non Communicable Disease Epidemiology, London School of Hygiene & Tropical Medicine, London, UK; Centre for Chronic Conditions and Injuries, Public Health Foundation of India, National Capital Region, India

**Keywords:** Alcohol, Depression, Suicidal attempt, Spouse, India, NCDs

## Abstract

**Background:**

Harmful effects of alcohol abuse are well documented for drinkers, and adverse effects are also reported for the physical and emotional well-being of family members, with evidence often originating from either drinkers or their families in clinic-based settings. This study evaluates intra-household associations between alcohol abuse in men, and depression and suicidal attempts in women, in community-based settings of Chennai, India.

**Methods:**

This community-based cross-sectional study of chronic disease risk factors and outcomes was conducted in n = 259 households and n = 1053 adults (aged 15 years and above) in rural and urban Chennai. The Alcohol Use Disorder Identification Test (AUDIT) score was used to classify alcohol consumption into ‘low-risk', ‘harmful’, ‘hazardous’ and ‘alcohol dependence’ drinking and the Patient Health Questionnaire (PHQ-9) score to classify depression as ‘mild’, ‘moderate’, ‘moderate-severe’ and ‘severe’. Multivariate logistic regression models estimated the association of depression in women with men’s drinking patterns in the same household.

**Results:**

A significant 2.5-fold increase in any depression (PHQ-9 ≥ 5) was observed in men who were ‘alcohol-dependent’ compared to non-drinkers (OR = 2.53; 95 % CI: 1.26, 5.09). However, there was no association between men’s drinking behavior and depression in women of the same household, although suicidal attempts approached a significant dose–response relationship with increasing hazard-level of men’s drinking (p = 0.08).

**Conclusion:**

No significant intra-household association was observed between men’s alcohol consumption and women’s depression, though an increasing (non-significant) trend was associated with suicidal attempts. Complex relationships between suicidal attempts and depression in women and male abusive drinking require further exploration, with an emphasis on intra-household mechanisms and pathways.

## Background

Alcohol is the third largest risk factor for premature mortality and disability globally, with 70 % of alcohol-related deaths occurring in developing countries [1]. Southeast Asian countries account for 25-33 % of males consuming alcohol worldwide [2] and rapidly rising trends are observed in women [3]. In India, nearly a third of men (32 %) aged 15–54 years report consuming alcohol with 9.4 % of them classified as ‘alcohol dependent’ [4]. The effects of alcohol abuse extends beyond the physical and mental health of the drinker [5] to include other members of the family, their household expenditures and various health outcomes such as mental health disorders and injuries [6].

Common mental health disorders, such as depression and anxiety, are a leading cause of disability in the global burden of disease [7] and there is ample evidence to suggest that women are at a higher risk for anxiety disorders and depression than men [8–11]. In both men and women, there is a potential bi-directional relationship whereby alcohol use disorders increase the risk of depression and vice versa through ‘self-medication’ to reduce stress or elevate mood [12–14], as well as a reciprocal causal relationship between depression and alcohol use disorders [12, 15]. Studies in low- and middle-income countries (LMICs) have found that the gender disadvantage and lack of autonomy puts women at higher risk for intimate partner violence, common mental disorders [16, 17] as well as suicide [18]. Socio-economic factors such as poverty, lack of economic activity, limited financial and social resources have also been found to be potential risk factors for poor psychological health of women, particularly in LMICs [19–21].

Recent data suggests that India’s rising suicide rates have led to the highest estimated number of suicides in the WHO South-East Asia Region [22]. At the individual-level, mental disorders, socio-economic status, chronic health problems and terminal illness are strong risk factors for suicide attempts in women [18, 23–25]. At the family-level, population-based studies have shown a strong relationship between alcoholism in men and the presence of intimate partner violence (IPV) [26–28] leading to depression and suicide in spouses, suggesting a potential intra-household mechanism for these outcomes in women [21, 29]. This is borne out through several studies in India, which also suggest that husband’s alcohol consumption is a trigger for domestic violence [30, 31–33] and may lead to depression and suicide of their spouses and other female family members in India [5, 21, 29, 34]. In India, as with other LMIC’s, the family/household units are often defined by multiple generations, extended families, constrained resources and traditional cultural norms whereby decision-making by one person can have major consequences on other members of the same household [3, 35]. Studies in high-income countries also demonstrate the influence of alcohol abuse of one family member on other members of the household [36, 37]. We conducted this study to evaluate the intra-household associations between hazardous drinking patterns in men with depression and suicidal attempts in female family members from rural and urban communities in Chennai, India.

## Methodology

### Study design

This study is a part of a multi-center, community-based cross-sectional study of non-communicable chronic disease (NCD) risk factors and outcomes (Chronic Disease Risk Factor [CDRF] Study). The data for this analysis stems from n = 1053 rural and urban participants residing in Sirudhavur (n = 734) and Thuraipakkam (n = 319), aged 15 years and above in and around Chennai. Persons residing for less than 6 months in a year or in a non-traditional household (e.g., female students sharing rooms in a single house) were excluded from the study as it was designed to evaluate families for the prevalence and interactions of individual- and household-level NCD risk factors and outcomes.

### Data collection

A structured, interviewer-administered questionnaire was conducted for each consenting participant in the household. Household-level data were collected on non-health expenditures, utilities, health insurance, exposure to indoor biomass fuel and monthly consumption of sugar, salt and oil. For adolescents and adults aged 15 years and above, individual-level data were captured on demographics, family and medical history, use of health care services, lifestyle habits (e.g., diet, physical activity, alcohol intake), anthropometrics (e.g., height, weight, waist, hip and mid-upper arm circumferences), outcomes (e.g., blood pressure, mental health, vision, disability) and suicidal attempts.

Demographic information included age in years, marital status (single, married, widowed, divorced), occupation (housework, skilled or unskilled manual, farming, student, professional/business owner, unemployed and other), religion (Hindu, Christian and Others including Sikh and Muslim) and education (illiterate or no formal education, primary education, secondary level, graduate and professional level). The General Physical Activity Questionnaire (GPAQ) was used to collect information on occupational, recreational and commute-related physical activity over the past one week, and hours of moderate and vigorous physical activity were summed across work and recreational activities. We also collected information on access/use of health care services (number of times visited doctor in past year), monthly household expenditures, number of household members and pain (degree of pain suffered in past four weeks as none, very mild, mild, moderate, severe and very severe). Self-reported medical history of major NCDs (eg, hypertension, diabetes, stroke, angina, kidney, cancer, asthma) was also collected for all consenting adult participants in the study.

### Alcohol abuse

The Alcohol Use Disorder Identification Test (AUDIT) is a 10-item questionnaire developed by the World Health Organization [38] to measure hazardous, harmful drinking and alcohol dependence and has been validated in international sittings to screen for alcohol use disorders [38–43]. It captures frequency of consumption, number of drinks per sitting, addiction/dependence, and interference of drinking on health and everyday activities. On a Likert scale of 1 to 5 (1 = Never, 2 = Less than monthly, 3 = Monthly, 4 = Weekly and 5 = Daily or almost daily), questions on heavy drinking (“How often have you had 6 or more drinks on one occasion?”), dependence (“How often have you needed a drink in the morning to get yourself going after a heavy drinking session?”) and remorse (“How often have you had a feeling of guilt or remorse after drinking?”) were scored. A score was generated from the sum of each of the 10 questions, whereby 1 = Non-drinkers and successively higher numbers reflects the severity of drinking. A score of 8–15 is classified as ‘low-risk drinking’ based on the frequency and quantity of alcohol intake. A score of 16–19 is classified as ‘hazardous drinking’ based on parameters of uncontrolled and increased drinking. A score of 20 and above measures ‘alcohol dependence’ on the account of guilt, blackouts and any alcohol-related injuries. The treatment modality differs for each group with simple advice offered for ‘low-risk drinkers’, brief counseling and continuous monitoring offered for ‘hazardous drinking’, and further diagnosis and evaluation for ‘alcohol dependence’ [38].

### Depression and Suicide

Depression was assessed using the Patient Health Questionnaire (PHQ-9), a 9-item questionnaire that is used in both clinical and research settings for screening purposes [44]. This brief measure parallels the major American Psychiatric Association’s Diagnostic and Statistical Manual of Mental Disorders (DSM-IV) criteria for the screening of major depression, and is a dual-purpose instrument that, with the same nine items, can establish provisional depressive disorder diagnoses as well as grade depressive symptom severity [45]. A summary score was calculated ranging from 0 to 27, where each of the 9 items is scored from 0 (“not at all”) to 3 (“nearly every day”). Cut points are marked at 5, 10, 15, and 20 representing thresholds for ‘mild’, ‘moderate’, ‘moderately severe’, and ‘severe’ depression, respectively [45], and scores less than 5 are considered ‘normal’. Scores ranging from 5 to 9 signify a potential mild level of depression and those persons are recommended for support and education to overcome depression. Scores of 10–14 refer to those with moderate depression, while scores of 15–27 refers to the presence of severe depression and such individuals are recommended for anti-depressant treatment and psychotherapy [46]. Due to smaller numbers of more severe forms of depression in our study population, we grouped ‘moderate’, ‘moderately severe’ and ‘severe’ depression into one category of ‘moderate-severe’ depression in our analyses. PHQ-9 scores were summed and classified according to ‘mild’ (PHQ-9 = 5-9), ‘moderate-severe’ (PHQ-9 = 10-27) and ‘any’ (PHQ-9 = 5-27) depression, which included all forms of depression combined. For all adults aged 15 years and above, a single question on suicidal attempts, ‘Did you ever attempt to commit suicide in the past 1 year?’ was also asked and evaluated as a yes/no binary outcome.

### Statistical analysis

Continuous variables were compared using the *t*-test at the alpha = 0.05 level while dichotomous variables were compared using the chi-squared test at the same level of significance. Multivariate regression models using robust standard errors to account for intra-household clustering were conducted for comparing female members’ depression (dichotomous yes/no, logistic regression) and PHQ-9 mean scores (continuous, linear regression) according to the drinking pattern of male drinkers (defined according to AUDIT summary score thresholds) in the same household. Models were adjusted for potential confounders and predictors such as age (continuous in years), education (illiterate or no formal education, primary level, secondary level, graduate/professional level), religion (Hindu, Christian and other), marital status (single, married, widowed/divorced), occupation of head of household (housework, skilled or unskilled manual, farming, student, professional/business owner, unemployed/other/not applicable), quartiles of physical activity, access/use of health care services (number of times visited doctor in past year), monthly household expenditures, number of household members and pain (degree of pain suffered in past four weeks as none, very mild, mild, moderate, severe and very severe). We evaluated the association between male drinking and female family members and not vice versa since less than 1 % of females reported drinking alcohol in our study population. For suicidal attempt, logistic regression models were conducted to compare whether any suicidal attempts in women were associated with men’s alcohol drinking patterns. STATA version 11 was used to conduct the analyses.

### Ethics

The study was approved by the Ethical Committees of participating institutes (Public Health Foundation of India, Voluntary Health Services) as well as the Indian Council of Medical Research, Health Ministry Screening Committee (No. 50/5/Indo-CVD/DP/2010-NCD-II). Informed written consent was obtained from all participants, who were ensured of confidential and secure data used for research purposes only. Participants had the right to refuse to answer any questions that they perceived to be uncomfortable.

## Results

Important demographic and lifestyle factors are presented for men and women, with and without depression (PHQ-9 ≥ 5 and PHQ-9 < 5, respectively) in Table 1. Nearly half of the population had completed their secondary education (49.4 %) and were employed in skilled/unskilled labour (46.8 %). Level of depression was observed to be high among males under the category of skilled/unskilled labour (62.9 %) whereas depression was comparatively higher among women involved in housework (42.1 %). Women who had no formal education (71.9 %) or primary education (55.2 %) had more depression than those with secondary education (42.3 %). Women with depression were more likely to be engaged in physical activity (27.3 %) but no such association was observed among men. Suicidal attempts were observed to be more common in those with depression amongst both men (6.6 %) and women (7.9 %). In comparison to women, alcohol consumption was highly prevalent among men. Level of depression was seen to be high (38.4 %) among those who consumed alcohol less than once a week in comparison to other drinking patterns. Across all the age groups, depression was more common in females compared to males (data not shown).

**Table 1:** Socio-demographic characteristics by gender and depression of the CDRF study population in Chennai, India

Variable		Males (n = 510)		Females (n = 543)	
	N (%)	No depression (PHQ-9 < 5) Mean (SD) or n (%)	Any depression (PHQ-9 ≥ 5) Mean (SD) or n (%)	No depression (PHQ-9 < 5) Mean (SD) or n (%)	Any depression (PHQ-9 ≥ 5) Mean (SD) or n (%)
Age		34.9 (0.65)		36.5 (0.66)	
Marital status					
Single	256 (24.4)	128 (35.9)	36 (23.7)	66 (26.7)	26 (8.9)
Married	693 (65.9)	226 (63.5)	113 (74.3)	160 (64.8)	192 (65.7)
Widowed/divorced	102 (9.7)	2 (0.6)	3 (1.9)	21 (8.5)	74 (25.3)
Occupation					
Housework	252 (24.1)	5 (1.4)	7 (4.6)	117 (47.6)	122 (42.1)
Skilled or unskilled manual	490 (46.8)	208 (58.6)	95 (62.9)	67 (27.2)	118 (40.7)
Farming	77 (7.4)	33 (9.3)	11 (7.3)	12 (49)	21 (7.2)
Student	128 (12.2)	61 (17.2)	18 (11.9)	35 (14.2)	12 (4.1)
Professional/business owner	64 (6.1)	35 (9.9)	10 (6.6)	6 (2.4)	12 (4.1)
Unemployed/Other	37 (3.5)	13 (3.7)	10 (6.6)	6 (3.7)	5 (1.7)
Religion					
Hindu	928 (88.5)	316 (89.0)	113 (88.1)	222 (90.2)	252 (86.9)
Christianity	104 (9.9)	31 (8.7)	18 (11.9)	19 (7.7)	35 (12.1)
Other (Muslim, Sikh)	16 (1.5)	8 (2.2)	0 (0.0)	5 (2.0)	3 (1.0)
Education					
Illiterate or no formal education	313 (30.0)	59 (16.8)	45 (30.0)	58 (23.7)	149 (51.6)
Primary	127 (12.2)	36 (10.2)	24 (16.0)	30 (12.2)	37 (12.8)
Secondary	515 (49.4)	212 (60.2)	65 (43.3)	135 (55.1)	99 (34.3)
Graduate/professional	87 (8.4)	45 (12.8)	16 (10.7)	22 (8.9)	4 (1.4)
Physical Activity (quartiles of number of hours per week of moderate- and vigorous-physical activity)					
1^st^ quartile (<=1)	167 (21.9)	50 (19.3)	21 (18.9)	56 (32.6)	40 (18.5)
2^nd^ quartile (>1-3)	181 (23.8)	60 (23.2)	28 (25.2)	42 (24.4)	50 (23.2)
3^rd^ quartile (>3-6)	202 (26.6)	61 (23.5)	34 (30.6)	40 (23.3)	67 (31.0)
4^th^ quartile (>6)	210 (27.6)	88 (33.9)	28 (25.2)	34 (19.8)	59 (27.3)
Suicidal attempts					
No	1011 (96.4)	353 (99.4)	142 (93.4)	244 (98.8)	269 (92.1)
Yes	38 (3.6)	2 (0.6)	10 (6.6)	3 (1.2)	23 (7.9)
Alcohol use					
Never	726 (69.5)	150 (42.1)	42 (27.8)	244 (100.0)	287 (99.3)
<=1 time/week	170 (16.3)	110 (30.9)	58 (38.4)	0 (0.0)	1 (0.4)
2-3 times/week	72 (6.9)	47 (13.2)	24 (15.9)	0 (0.0)	1 (0.4)
4+ times/week	76 (7.3)	49 (13.8)	27 (17.9)	NIL	NIL
AUDIT score					
Non-drinker (0–7)	726 (69.6)	150 (42.2)	42 (27.8)	244 (100.0)	287 (99.3)
Low risk (8–15)	95 (9.1)	66 (18.6)	28 (18.5)	NIL	NIL
Harmful, hazardous (16–19)	69 (6.6)	45 (12.7)	23 (15.2)	0 (0.00)	1 (0.4)
Alcohol dependence (20–45)	153 (14.7)	94 (26.5)	58 (38.4)	0 (0.00)	1 (0.4)

Individual-level associations revealed that in men, ‘alcohol dependent’ drinkers (AUDIT score of 20–45) were more than 2.5 times likely (OR = 2.53; 95 % CI: 1.26, 5.09) to have any depression (PHQ-9 scores = 5-27) than non-drinkers (AUDIT score of 0–7; Table 2). Similar magnitudes of association were observed for ‘mild’ (PHQ-9 score of 5–9; OR = 1.53; 95 % CI: 0.86, 2.71) or ‘moderate-to-severe’ depression (PHQ-9 10–27; OR = 1.91; 95 % CI: 0.89, 4.07; Table 2).

**Table 2:** Association^a^ of depression (from PHQ-9 thresholds) with alcohol drinking (AUDIT score) among men in Chennai, India

	Mild depression (PHQ-9 = 5-9)						Moderate-severe depression (PHQ-9 = 10-27)						Any depression^b^ (PHQ-9 = 5-27)					
Drinking pattern (AUDIT score)	Age- adjusted OR	95 % CI	P value	Multi- variate OR^c^	95 % CI	P value	Age- adjusted OR	95 % CI	P Value	Multi-variate OR^c^	95 % CI	P value	Age-adjusted OR	95 % CI	P value	Multi- variate OR^c^	95 % CI	P value
Non-drinkers (score^d^: 0–7)	Ref.			Ref.			Ref.			Ref.			Ref.			Ref.		
Low risk (score: 8–15)	1.31	(0.68, 2.51)	0.41	1.48	(0.65, 3.33)	0.34	1.29	(0.50, 3.29)	0.58	1.43	(0.37, 5.57)	0.59	1.31	(0.73, 2.33	0.35	1.50	(0.72, 3.15)	0.27
Harmful, hazardous (score: 16–19)	1.37	(0.66, 2.84)	0.38	1.83	(0.71, 4.69)	0.30	1.33	(0.50, 3.56)	0.56	1.98	(0.48, 8.08)	0.33	1.37	(0.73, 2.59)	0.32	1.88	(0.81, 4.34)	0.13
Alcohol dependence (score: 20–45)	1.53	(0.86, 2.71)	0.14	2.11	(0.95, 4.66)	0.06	1.91	(0.89, 4.07)	0.09	3.88	(1.18, 12.77)	0.02	1.66	(1.01, 2.72)	0.04	2.53	(1.26, 5.09)	0.00

^*a*^Based on logistic regression models

^b^Any depression is defined as a composite variable for mild, moderate, moderately severe and severe depression

^*c*^Adjusted for age, occupation, monthly household expenditures, physical activity, number of household members, pain in past 4 weeks, number of self-reported NCD’s (eg, hypertension, diabetes, stroke, angina, kidney, cancer, asthma)

^*d*^AUDIT score

When we evaluated intra-household associations between alcohol drinking in men, and depression in women of the same household however, we observed no significant association or dose–response relationship (Table 3). We also evaluated associations with PHQ-9 summary scores (mean (SD) = 3.56 (0.17) for men and 6.16 (0.22) for women), and found higher scores for women living with ‘alcohol dependent’ drinkers compared to non-drinkers (mean PHQ-9 score of 7.0 and 6.14, respectively), but the difference was not statistically significant after adjusting for important confounders in multivariate models (p = 0.58; data not shown). We conducted a sub-analysis to consider stronger effects in spouses only, but our conclusions remained the same (RR = −0.18; 95 % CI: −0.68, 0.31; data not shown).

**Table 3:** Intra-household association^a^ of depression in women and alcohol drinking (AUDIT score) among men in Chennai, India

	Mild depression (PHQ-9 = 5-9)						Moderate-severe depression (PHQ-9 = 10-27)						Any depression^b^ (PHQ-9 = 5-27)					
Drinking pattern (AUDIT score)	Age-adjusted OR	95 % CI	P value	Multi-variate OR^c^	95 % CI	P value	Age-adjusted OR	95 % CI	P value	Multi-variate OR^c^	95 % CI	P Value	Age- adjusted OR	95 % CI	P value	Multi-variate OR^c^	95 % CI	P value
Non-drinkers (score^d^: 0–7)	Ref.			Ref.			Ref.			Ref.			Ref.			Ref.		
Low risk (score: 8–15)	1.97	(0.99, 3.90)	0.05	1.80	(0.73, 4.44)	0.19	1.18	(0.55, 2.49)	0.66	1.15	(0.41, 3.23)	0.78	1.88	(1.01, 3.49)	0.04	1.64	(0.71, 3.78)	0.24
Harmful, hazardous (score: 16–19)	1.14	(0.50, 2.61)	0.75	1.03	(0.38, 2.79)	0.95	1.66	(0.72, 3.81)	0.22	1.49	(0.51, 4.30)	0.46	1.40	(0.69, 2.86)	0.34	1.06	(0.44, 2.53)	0.88
Alcohol dependence (score: 20–45)	1.11	(0.61, 2.03)	0.71	0.99	(0.46, 2.12)	0.98	0.69	(0.34, 1.37)	0.29	0.82	(0.32, 2.09)	0.67	1.01	(0.57, 1.76)	0.97	0.85	(0.42, 1.72)	0.65

^*a*^Based on logistic regression models with correlated errors accounting for intra-group correlation of depression in women of the same household

^b^Any depression is defined as a composite variable for mild, moderate, moderately severe and severe depression

^*c*^Adjusted for age, occupation, monthly household expenditures, physical activity, number of household members, pain in past 4 weeks, number of self-reported NCD’s (eg, hypertension, diabetes, stroke, angina, kidney, cancer, asthma)

^*d*^AUDIT score

We evaluated suicidal attempts in the last year and found an increasing, non-significant trend with increasing hazard levels of men’s alcohol drinking (Table 4). The association approached borderline significance when we focused on wives (p = 0.08) only. The number of men who reported a suicidal attempt in the past year was too small to conduct a separate analysis in this sub-group.

**Table 4:** Intra-household association^a^ of suicidal attempts in women and alcohol drinking (AUDIT score) in men in Chennai, India

	All females in households (n = 543)		Wives only (n = 299)	
Drinking pattern (AUDIT score)	Multivariate OR^b^	95 % CI	Multivariate OR^b^	95 % CI
Non-drinkers (score^c^: 0–7)	Ref.		Ref.	
Low risk (score: 8–15)	0.97	(0.12, 7. 46)	1.94	(0.15, 24.26)
Harmful, hazardous (score: 16–19)	3.71	(0.66, 20.75)	4.30	(0.40, 46.09)
Alcohol dependence (score: 20–45)	4.01	(0.81, 20.75)	6.55	(0.75, 56.77)
Test-for-trend	*p = 0.10*		*p = 0.08*	

^*a*^Based on logistic regression models

^*b*^Adjusted for age, occupation, monthly household expenditures, physical activity, number of household members, pain in past 4 weeks, number of self-reported NCD’s (eg, hypertension, diabetes, stroke, angina, kidney, cancer, asthma)

^c^AUDIT score

## Discussion

In this study, nearly two thirds of men reported drinking alcohol, in whom we observed a significant dose–response relationship with depression (PHQ-9). At the household level, we did not observe a significant association with depression (PHQ-9 score) in females of the same household, although there was an increasing, non-significant trend associated with suicidal attempts. Studies [5, 34] on the link between spousal alcohol use and mental health of women is mediated by many factors including intimate partner violence, chronic physical problems [46] and women’s own alcohol use [17, 21, 47, 48].

At the individual-level (associations between depression and drinking in men), our results are consistent with the evidence, and possible explanations can include difficulties in family and social life, employment, legal troubles, compromised physical health and genetic factors [49, 50]. At the household-level (associations between depression and suicidal attempts in women and drinking in men), our findings are similar to those of Schuckit and colleagues [51] who found no association between psychiatric disorders among female spouses of male alcohol abusers, and to other studies in which spouses of male alcohol abusers present with fewer psychiatric symptoms than spouses with lower consumption levels due to certain adaptive activities that provide stability in stressful situations [52, 53]. Though non-significant, the magnitude of associations for depression in our study were greater for women living with ‘low-risk’ drinkers than those living with more hazardous drinkers (Table 3). Studies indicate that the relationship between alcohol use and partner’s depression is influenced by a number of psychosocial risk factors [16] such as spouse’s attitude towards drinking, alcohol-related marital problems, social support, resilience and IPV [54, 55]. Although intra-household evaluations did not reveal any trends or association for depression in our study, an increasing trend approached significance for suicidal attempts (Table 4). While systematic reviews from developed countries suggest that persons with mental disorders constitute the majority of persons with suicidal ideation and suicidal attempts [56, 57], the proportion may be lower in developing countries, so understanding multiple pathways for suicide is critical [58].

The evaluations in this study assume potential intra-household pathways between hazardous drinking patterns in men and depression and suicidal attempts in women. Factors involved in intra-household pathways between alcohol consumption and mental health, may include social and environmental factors [59] and bi-directional models [15, 60, 61]. A recent study of problematic drinkers also demonstrated that several factors and circumstances can give rise to both; for example, job loss, family problems, financial stress or other addictions may be potential triggers for depression, even in the absence of a detrimental role of alcohol and may also simultaneously lead to consumption of alcohol [59]. A potential bi-directional relationship between alcohol use disorders and mental depression may exist such that each disorder increases the risk of the other disorder simultaneously [15, 60]. The specific underlying mechanisms that give rise to such associations are unclear, particularly in LMIC settings, where the household unit may have different structural characteristics and dynamics. Interventions to address these behavioral and health problems, both at the individual- and household-level, requires research and evidence to unpack and identify the relevant mechanisms of interest.

One strength of this study is that data were collected on all members of the household and therefore, information on alcohol use came from the drinkers themselves and questions related to mental health from spouses and other members - there were no proxy respondents - using standardized, validated tools. This enabled a more objective evaluation of intra-household effects of alcohol abuse and offers a unique contribution to the literature from a community-based setting in a developing country. One limitation of the study is that we did not measure domestic violence or inter-personal violence (IPV), which is one important mediator of the association between alcohol abuse in men and depression in other members of the household [62]. Our self-reported responses for PHQ-9 could not be validated against a gold-standard diagnostic or clinical confirmation (such as CIDI Composite International Diagnostic Interview [63] and Beck depression Inventory (BDI) [64]), although PHQ-9 is increasingly used in community-based low-resource settings as an initial screening tool to flag persons for referral, counseling and/or follow-up [65–69]. Moreover, a meta-analysis by Gilbody, Richards, Brealey, and Hewitt (2007) showed that the pooled sensitivity of PHQ-9 as a diagnostic tool for major depression was 0.80 (95 % confidence interval [CI]: .71-.87) and specificity was 0.92 (95 % CI: 0.88 0.95) [70]. Given the sensitive nature of the question, there is likely to be under-reporting of information on suicidal attempts, and therefore the sample size is very small for detecting significant associations. Another limitation is the inability to infer causality and/or direction of the relationship, as this is a cross-sectional study design.

## Conclusion and policy implications

India has the highest suicide rates and it is the leading cause of death among young and elderly in the South-east Asia region [18, 22]. Considering the magnitude of the problem, it is important to study the mulifactorial mechanisms related to social, biological and lifestyle determinants as well as potential household-level pathways. Evidence indicates alcoholism and IPV as two potential risk factors for suicide in women in LMICs [62]. Policies and strategies aimed at improving the mental health status of women should consider the association with alcohol use (and potentially IPV) by their partners, as well as other household-level influences. Routine enquiries regarding suicidal ideation, thoughts or behavior amongst spouses of men who have drinking problems and amongst women who complain of IPV, is one potential strategy. Similarly, a broader household-based approach can be considered in which the mental health of family members can be assessed alongside services designed for the alcohol abuser. The recently released National Mental Health Policy of India [71] by the government of India also acknowledges the need to address social determinants of mental health such as poverty, environmental issues and education to improve the country’s overall health status.
